# Perches as Cooling Devices for Reducing Heat Stress in Caged Laying Hens: A Review

**DOI:** 10.3390/ani11113026

**Published:** 2021-10-21

**Authors:** Jiaying Hu, Yijie Xiong, Richard S. Gates, Heng-Wei Cheng

**Affiliations:** 1 Department of Animal Science, Purdue University, West Lafayette, IN 47907, USA; hu165@purdue.edu; 2 Departments of Animal Science, and Biological Systems Engineering, University of Nebraska-Lincoln, Lincoln, NE 68583, USA; yijie.xiong@unl.edu; 3 Departments of Animal Science, and Agricultural and Biosystems Engineering, Egg Industry Center, Iowa State University, Ames, IA 50011, USA; rsgates@iastate.edu; 4 Livestock Behavior Research Unit, USDA-ARS, West Lafayette, IN 47907, USA

**Keywords:** heat stress, behavior, production, physiology, immunology, health, welfare, hen

## Abstract

**Simple Summary:**

High ambient temperature is a critical environmental challenge to the egg industry worldwide. Laying hens that are under heat stress are unable to maintain a balance between body heat production and heat loss, leading to hyperthermia, which substantially disturbs physiological homeostasis and consequently reduces all parameters of production performance, e.g., egg production, egg quality, feed intake, feed efficiency rate, and longevity—ultimately causing substantial economic losses. To alleviate these deleterious effects, the egg industry and poultry scientists are working towards developing cooling methods to prevent heat stress. This review summarizes our recent discoveries that perches can be used as cooling devices to avoid or reduce heat stress detrimental effects on hen production, health, and welfare. Our results provide a novel strategy: perches, one key furnishment in cage-free and enriched colony facilities of modern laying hens, could be modified as cooling devices to improve hen thermal comfort during hot seasons.

**Abstract:**

Heat stress is one of the most detrimental environmental challenges affecting the biological process and the related production performance of farm animals, especially in poultry. Commercial laying hens have been bred (selected) for high egg production, resulting in increased sensitivity to heat stress due to breeding-linked metabolic heat production. In addition, laying hens are prone to heat stress due to their inadequate species-specific cooling mechanisms resulting in low heat tolerance. In addition, hens have no sweat glands and feathering covers almost their entire body to minimize body heat loss. The poultry industry and scientists are developing cooling methods to prevent or reduce heat stress-caused damage to chicken health, welfare, and economic losses. We have designed and tested a cooling system using perches, in which chilled water (10 °C) circulates through a conventional perch passing through the layer cages to offer the cooling potential to improve hen health, welfare, and performance during acute and chronic periods of heat stress (35 °C). This review summarizes the outcomes of a multi-year study using the designed cooled perch system. The results indicate that conducting heat from perching hens directly onto the cooled perch system efficiently reduces heat stress and related damage in laying hens. It provides a novel strategy: perches, one key furnishment in cage-free and enriched colony facilities, could be modified as cooling devices to improve thermal comfort for hens during hot seasons, especially in the tropical and subtropical regions.

## 1. Introduction

A high ambient temperature is one of the most deleterious environmental stressors affecting the commercial poultry egg industry worldwide. Notably, climate change over the past decades has resulted in more hot days with unexpected heat waves [1,2,3,4], which is a considerable challenge to all exposed biological subjects [5], including chickens. Hens’ abilities to lose body heat are limited due to feathering, the absence of sweat glands, and a relatively high metabolic rate resulting from breeding for high egg production [6]. Akin to mammals, their core body temperature is precisely controlled within a relatively narrow range. A hen can tolerate and adapt to ambient temperatures up to approximately 25 °C (77 °F), and temperatures above this level activate various pathways for thermoregulation to cope with thermal stress. Hens that are unable to adapt to heat stress (HS) experience greater heat gain than heat loss, leading to hyperthermia (increased body core temperature), which causes thermal damage in various organs and tissues [7] and eventually leads to death [8]. For those hens who survive high temperatures, HS negatively affects their physiological, immunological, and intestinal functions, as well as reducing all economically important production traits such as egg production, egg weight, shell strength and thickness, and feed intake and efficiency, leading to substantial economic losses [9,10,11]. Currently, egg producers struggle to combat HS during hot summer seasons; there has been a recent upswing in both the length and frequency of heat waves, as measured by two or more days, with an ambient daily minimum temperature that exceeds the 85th percentile of historical summer conditions in many egg-producing areas [12].

Commercial laying hens have been intensively selected for greater egg production and feed efficiency. Compared to their ancestors—jungle fowls lay 4–6 eggs per year—a laying hen produces more than 300 eggs a year [13]. The genetic selection of high production in egg-laying type chickens has caused layers to become more sensitive to stressors, such as HS, due to selection-caused physiological changes and related metabolic disorders (such as high metabolic heat production and oxidative stress). The more productive lines of layers are more likely affected by elevated ambient temperatures [14,15]. Management practices that expose hens to repeated or chronic stress can affect their ability to adapt to presented stressors or respond to new stressors (allostatic load, i.e., accumulated adverse effect on the body when an individual is exposed to multiple combined, repeated, or prolonged chronic stress), such as HS and induced molting. Induced molting (traditional fasting molt and recently developed non-feed withdrawal molt) is a management strategy to rejuvenate hens’ reproductive systems at the end of the laying cycle, and then bring the flock into a second laying cycle based on the egg market demand and reduce bird production cost per dozen eggs [16]. Fasting molt also changes gut microbiota composition, reducing *Lactobacillus* genus and increasing pathogenic bacteria such as *Enterococcus cecorum* and *Escherichia coli* in aged laying hens [17], consequently suppressing immunity. Non-feed withdrawal molting by providing low-energy ingredients (such as wheat middlings, corn distillers dried grains with solubles, or corn diets) may negatively affect laying hens as low energy diets may not lead to satiated feeding, even though the crop and proventriculus may be distended following ingestion [18]. With climate change-related prolonged hot weather, molting may be unavoidably induced during summer under high ambient temperatures to meet the market needs or to avoid interruption in the egg market due to disease outbreaks. Hen health and welfare may be further affected by accumulated (or combined) adverse effects of HS and induced molt.

To alleviate the negative effects of HS on laying hens, various methods have been employed that can be broadly categorized into three approaches: nutritional strategies (feed manipulations, electrolyte supplementations, antioxidant enzymes, and prebiotics, probiotics, and synbiotics), genetic strategies (high thermo-tolerance strains), and management strategies (control flock density and providing shelter, shade, cooling fans, ventilation design, and sprinklers) [4,19,20,21,22,23,24,25]. Tunnel ventilation systems, as an example, are common in broiler production and increasingly in newer US egg production facilities. This method brings high-velocity airflow over and through the flock to substantially increase sensible heat loss via convection, effectively lowering the ambient temperature. A limitation of this method is that as ambient conditions approach bird temperature, there is limited potential for convective heat loss. Evaporative cooling pads and fogging are sometimes used in hen barns and can effectively reduce indoor temperature [26], especially in drier climates. All the currently available cooling methods have certain disadvantages. For example, evaporative cooling, as well as tunnel ventilation, reduce house temperature but can be seriously compromised by the challenge of providing sufficient cooled air to the birds when in cages, and by the development of a longitudinal building temperature gradient as the heat produced by hens raises the air temperature from inlet to outlet. In addition, fogging, misting, and evaporative cooling all increase the humidity of housing facilities, which interferes with the bird’s ability to dissipate sensible heat through panting, a thermoregulatory behavior that releases body heat through accelerated respiration [27]. Furthermore, increased humidity leads to wet manure and litter, overgrowth of bacteria, and excessive ammonia levels—a health concern to chickens and caregivers [28,29]. Currently, most laying hens in the United States (approximately 76.4%, i.e., 257.1 million hens) are housed in conventional cages [13].

Laying hens are highly motivated to perform roosting behaviors when provided access to perches [30,31]. Perches installed in caged and cage-free environments increase hen comfort levels and skeletal health by providing places for roosting and related activities [32,33]. Installed perches have the potential to serve as a cooling device by conducting body heat to the cooled perches during roosting. The blood flow rate through the chicken’s feet is dependent on environmental temperatures and rises with increased ambient temperatures (2.2 mL/min at thermoneutral temperatures vs. 5.4 mL/min at 36 °C) [34]. Approximately 25% of the body heat of a chicken can be lost via its feet through a unique anatomical feature: the feet and shanks are unfeathered with little muscle tissue and are richly vascularized [35]. 

Currently, the U.S. egg industry is moving away from conventional cage production systems, the way Europe and Canada have. To meet some natural behavioral needs of laying hens, an enriched cage system with furnishments such as perches, nests, and a scratchpad (or sand bath area) was developed in the early 2000s. Perches in enriched cages, enriched colonies, and cage-free aviaries could be modified as cooling devices to improve hens’ thermal comfort, health, and welfare. Previous studies have reported that cooled perches improve broiler welfare and growth performance, including decreased mortality, improved feed efficiency, and body weight under HS [36,37,38,39,40,41]. However, limited studies have been conducted in laying hens. 

For this reason, we have designed and tested a cooling system in which chilled water (10 °C) is circulated through conventional perch pipes passing through layer cages. The outcomes support our hypothesis that conductive cooling directly from hens to cooled perches efficiently reduces HS and related damages in laying hens. These results provide a novel strategy: Perches, one key furnishment in enriched cages and cage-free facilities, can be modified as cooling devices to improve hen thermal comfort during hot summer seasons, especially in the tropical and subtropical regions.

## 2. Cooled Perch Design and Experimental Treatments

### 2.1. Engineering Design of the Thermal Perch System

**Figure 1:**
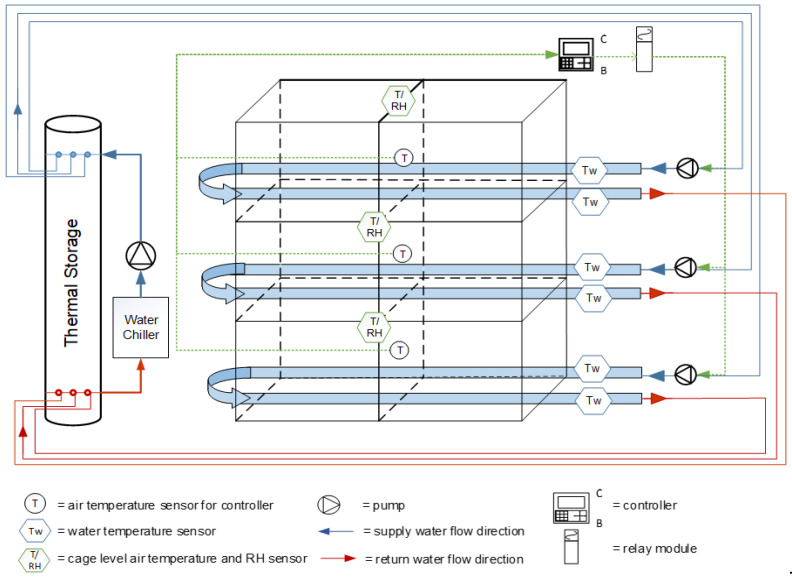
Schematic of the cooled perch system and instrumentation. Two systems were fabricated. Each system consisted of three loops (top, middle, and bottom) and each loop was individually operated by a pump that drew chilled water from a thermal storage manifold. The thermal storage was cooled by an independent loop consisting of a pump that continuously circulated water between the manifold and a water chiller (10 °C). Each loop pump was individually thermostatically controlled based on the air temperature within the cage. Instrumentation included inlet and return line water temperatures, cage air temperatures, and relative humidity (RH) (Modified from [42,43]).

To resemble commercial conventional cage egg production facilities, a tier cage system was used in our study. Each cooled perch (CP) unit consisted of a three-tier (top, middle, and bottom) bank with two cages (76 cm × 52 cm × 18 cm/cage) per tier. In each tier, two pieces of galvanized perch pipe (33.8 mm O.D. and 28.5 mm I.D.) were passed through the two cages, functioning as one supply pipe and one return pipe to form a complete perch loop with two 90° elbows (Figure 1) [42]. Each perch loop was approximately 6.1 m in total length, counting all fittings, with 3.0 m of usable length for the hens. A water chiller (model ER-101y, ELKAY Manufacturing Co., Oak Brook, IL, USA) had its thermostat set at 10 °C during operation. Chilled water was pumped to a thermal storage manifold, then independently pumped (pump model: 006-B4-15 Cartridge Circulator, Taco Inc., Cranston, RI, USA) to each circulating loop. All the exposed sections of each loop outside the cages and the manifold were insulated with polyethylene pipe insulation (0.033 W/m-K) to conserve energy and minimize condensation potential. The pumps were automatically turned on through a central controller when the cage air temperature reached 25 °C.

During the multi-year experiment (a four-year study, from 2014–2017), the air temperature of the research facility was controlled by fan ventilation with a continuously operating poly-tube distribution system. A wireless data acquisition system was developed for monitoring and collecting the room, cage, and perch loop water temperatures (ZW series, Onset Computer Co., Bourne, MA, USA) at 1 min intervals throughout the entire experiment. The temperature of hens’ feet was also detected using an infrared image camera (Model T440, FLIR Systems, Inc., Wilsonville, OR, USA). In addition, all the sensors were checked daily via a wireless delivery system and calibrated within the application range against a T/RH device certified by the National Institute of Standards and Technology when needed.

**Figure 2:**
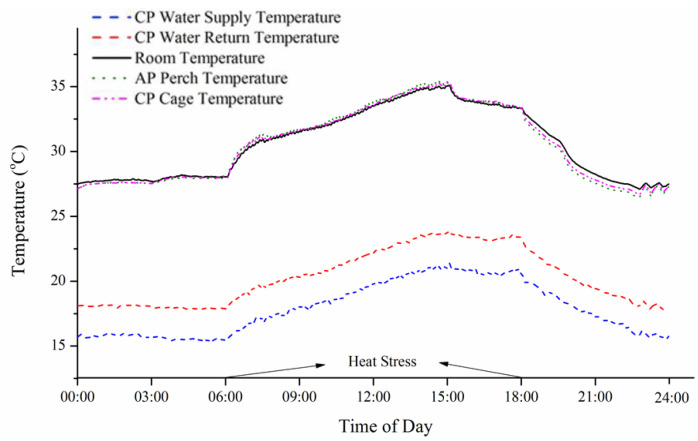
An example of temperatures recorded for 24 h during a heat episode (0600 to 1800 h). Notes: Supply/return temperatures maintained a 2–3 °C difference. Return temperature was warmer than supply temperature. Ap, air perch; CP, cooled perch; and NP, non-perch. Copied from [43].

**Figure 3:**
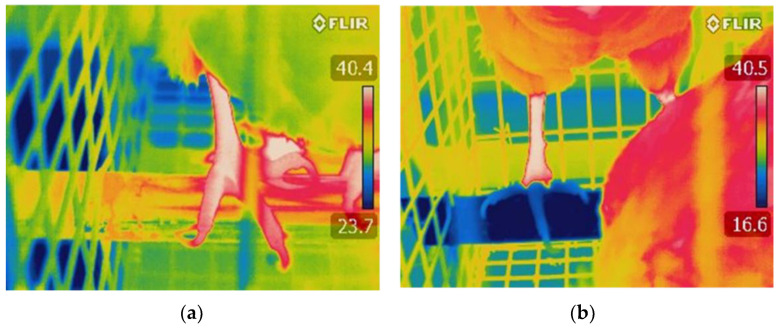
Examples of infrared thermal images (FLIR T440, FLIR Systems, Inc.; the camera was set at an emissivity of 0.95) of the feet temperature of hens standing on: (**a**) an air perch; and (**b**) a cooled perch. Notes: Compared to air perch hens, cooled perch hens had much colder feet.

The system worked well, as evidenced by a one-day snapshot of the temperature profile in a CP bank tier (Figure 2), indicating that the return-loop water temperature was substantially higher than that of supply-loop water. In addition, CP hens during the first cycle of lay had lower rectal temperatures at the end of both cyclic heating episodes as compared to non-perch (NP) and air perch (AP) hens [44]. This demonstrates that cooled perches allow for conductive heat loss from hens’ feet to the chilled water (Figure 3) to improve their thermoregulatory capability, thus maintaining body temperature and increasing thermal comfort, health, welfare, and production during hot weather. Increased rectal temperature, as an indicator of core body temperature, has been detected in laying hens and broiler chickens subjected to acute and chronic HS [45,46,47]. Similar to our findings, lower rectal temperatures were reported in broiler chickens provided CP compared to controls with access to regular perches [40]. The results support our hypothesis that conductive heat loss from hens’ feet to CP will supplement latent heat loss from panting so that hen comfort will be further enhanced during hot weather.

Engineering design criteria were extrapolated from this study. Xiong et al. [42] demonstrated that the net average daily perch heat gain was approximately 2334 W, about 256 W m^−1^ perch length, or 43.2 W per hen housed. These values are based on the system operating with 12 h day and 12 h night air temperatures of 35 °C and 28 °C, respectively, and an average daily loop inlet water temperature of 20 °C during HS. This information provides useful insights for future thermal perch system designs. For instance, controlling the inlet water temperature to 10 °C with the same temperature schedule would increase the average daily net perch heat gain by approximately 1.9 times, thus enabling greater heat conduction between hens’ feet and the chilled water, but would need to be evaluated against a “too cold” perch that might inhibit its use.

### 2.2. Experimental Treatments and Birds

Three hundred and twenty-four Hy-Line W-36 White Leghorns were used in this multi-year experiment. Hens from day-old until 110 weeks of age went through three chronic HS episodes from weeks 21 to 35 and 73 to 80 during the first egg-laying cycle, and weeks 82–110 with induced non-feed withdrawal molting from weeks 85 to 88 during the second egg-laying cycle.

Day-old chicks were reared in grower cages with (n = 20) or without perches (n = 10) at a temperature- and lighting-controlled grower facility. Each cage had 13 birds (stocking density of 286 cm^2^/pullet). At 17 weeks of age, the pullets were transferred to a temperature- and lighting-controlled layer facility and were randomly assigned to 36 nine-bird cages in six banks based on the rearing conditions (with or without perches) during the grower phase. Bird space allowance conformed to current industry guidelines (stocking density of 439 cm^2^/hen) [13].

**Figure 4:**
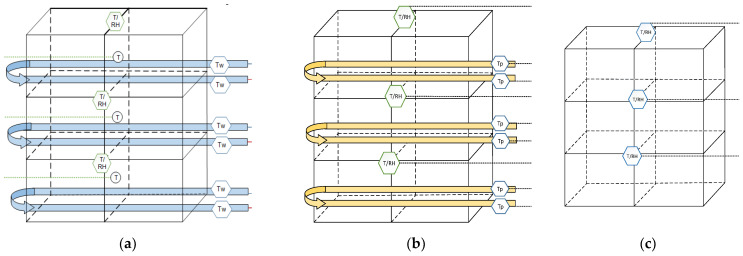
Cage bank design for the 3 treatments of: (**a**) cooled perch (CP) cages; (**b**) air perch (AP) cages; and (**c**) control cages with non-perch cages (NP). Two perches were installed parallel to each other in each row of a cage bank, forming a complete loop. For CP cages, a manifold was used to supply the perch loops with chilled (10 °C) water (Figure 1). Each CP loop was independently controlled by a thermostat-activated water pump, activated when the cage air temperature exceeded 25 °C (Figure modified from [42,43]).

The six cage banks were semi-randomly assigned into 1 of 3 treatments (Figure 4): CP (with circulating chilled water), AP (without circulating chilled water, positive control), and NP (negative control). The perches of each cage provided 16.9 cm of perch space per hen, which was adequate for all 9 hens to roost simultaneously [13].

During HS episodes, the room air temperature was raised to 35 °C for 12 h (0600–1800) and then went down to thermoneutral condition (1800–0600) daily, using furnaces from weeks 21 to 35 (2014 summer) and weeks 73 to 80 (2015 summer), as well as weeks 85 to 88 (2015 fall), plus non-feed withdrawal molting. During the unstressed periods, the ambient temperature was maintained at a thermoneutral condition, 20–25 °C, based on natural temperature fluctuations. Room temperature and relative humidity data were collected with data loggers at 1 min intervals throughout the experimental period.

All hens were fed regular layer diets based on their growth phases prior to the induced molt. At week 85, a low-energy, non-fast molt diet was used for a 28-day trial, then returned to the regular layer diet [48,49]. Food and water were provided for ad libitum consumption. Daily lighting was 16 L:8 D, except during molting, when it was restricted to 8 L:16 D.

## 3. Thermal Perch System Improves Adaption to Heat Stress in Laying Hens

### 3.1. Behavioral Adaption

The behavioral response (a specific instance of behavior), in general, is an important defense mechanism an organism uses for acting and interacting with the environment to maintain a balance in the internal body system. For instance, similar to most animals, hens typically use coping behaviors such as eating less, drinking more, panting (hyperventilation—heat loss accomplished by the evaporation of moisture from the buccal cavity and upper respiratory tract), wing spreading (rearranged feathers to positions that facilitate heat loss), and seeking cooler areas when confronted with HS. Among these heat stress-associated behaviors, panting is the primary method used by birds to dissipate internal heat [50]. However, excessive panting causes birds to expire increased amounts of carbon dioxide and develop metabolic alkalosis, a severe disruption of acid-base balance [51,52,53]. These negative effects of HS on hen behaviors can be reduced by providing the CP system [42,54].

**Figure 5:**
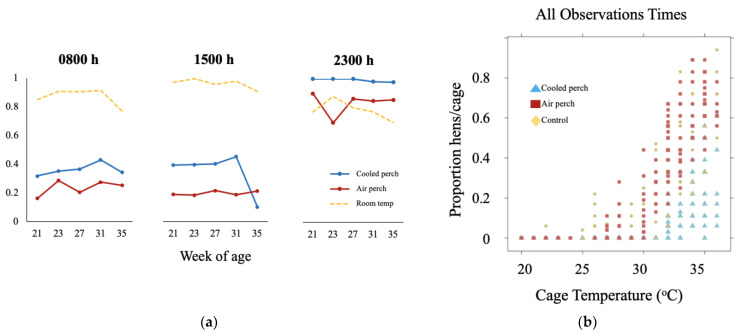
The effects of cooled perches on heat stressed-induced behaviors in caged laying hens from 21 to 35 wk of age. (**a**) perching behavior; and (**b**) proportion of hens per cage perching at different room temperatures.

In our studies, the average proportion of hens perching, panting, and wing spreading was determined via live observations conducted for one day every other week at 0800–0900 h, 1500–1600 h, and 2300–2400 h during the HS episodes. Perch use was always higher in CP versus AP cages (*p* < 0.001) (Figure 5a). The number of hens perched on the CP changed with the pattern of the daily HS schedule (HS was from 0600–1800 h): perching quickly increased to a peak by 11 am with the temperature increased in the morning and declined with decreased room temperature. Fewer CP hens (*p* < 0.001) panted than AP or NP birds at all time points. Wing spreading occurred in fewer CP hens (*p* < 0.001) than AP hens in the morning, and fewer CP hens than NP hens at all time points (0800–0900 h, 1500–1600 h, and 2300–2400 h). Day effects, when observed, seemed to be driven by differences in temperature profiles on individual observation days. Overall, across weeks, an average of 50% NP and AP hens began panting at 32 °C, but an average of 21.8% CP hens was panting at peak temperature (approx. 35 °C) (Figure 5b). The results indicate that access to CP increased perch use and reduced the proportion of hens performing thermoregulatory behavior (such as panting) during HS exposure. The CP system improved behavioral homeostasis in heat-stressed laying hens.

### 3.2. Mortality and Production Traits

In our study, hen mortality and egg production were recorded daily. Feed utilization, egg weight, and eggshell quality traits were measured at 4-week intervals during the HS episodes and at 8-week intervals during thermoneutrality [43]. Compared to NP hens, CP hens had lower cumulative mortality (*p* = 0.02. Table 1). CP hens also had higher egg production (*p* < 0.01) and feed usage (*p* < 0.05), without effects on the number of marketable eggs, compared to both AP and NP hens. In addition, eggs from CP hens had overall greater weights (*p* < 0.01) and breaking force (*p* < 0.01), without effects on eggshell thickness (*p* = 0.44). These results indicate that CP effectively mitigates the negative effects of HS on egg production performance, eggshell traits, and the livability of laying hens. Providing the CP system likely assists hens with adapting to HS by conducting body heat from their feet to the perches. Compared to both AP and NP hens, less effort is needed for CP hens to maintain thermal homeostasis and related metabolism through other methods rather than eating less (limiting energy intake from the feed) and panting (increasing evaporation heat loss).

### 3.3. Physical Conditions

The plumage condition was determined at five different body parts (breast, back, wings, vent, and tail areas) on the last day of the second heating episode (80 wk of age). The average feather condition was not affected by the provision of perches (Table 2). Foot health was determined by evaluating both foot pads and all toes for hyperkeratosis. The number of broken claws and eight nail lengths of each hen were also measured. At 80 wk of age, there was no treatment effect on hyperkeratosis (*p* > 0.05). Similarly, toenails were not affected by the perch installations (*p* > 0.05). Interestingly, more broken toenails were found in AP hens than CP hens (*p* < 0.05) but not NP (*p* > 0.05, Table 2). Hens with access to CP may have spent longer periods of time remaining on the perches to avoid the hot room temperature by increasing heat conduction, while the AP hens may be continuously switching to find a cooler area during heat exposure, leading to a higher incidence of broken nails. Our results indicate CP installations did not compromise overall plumage conditions and foot health in laying hens.

### 3.4. Physiological and Immunological Changes

Body temperature is regulated by the thermoregulatory center within the hypothalamus via the major thermoregulation system, i.e., the hypothalamic (thyrotropin-releasing hormone, TRH)-pituitary (thyroid-stimulating hormone, TSH)-thyroid (thyroxine, T4) system, to initiate heat regulations. The most hormone produced by the thyroid gland is T4 (thyroxine, approximately 94% of hormone released by the thyroid gland). T4 must be converted to T3 (3,5,3′-triiodothyronine), the active form of thyroid hormones, for proper thyroid function to initiate a cascade of biochemical reactions within various cells and thereby to play an important role in energy metabolism and thermogenesis [55,56]. Generally, warm-blooded (or endothermic) animals’ immediate coping behavior, when presented with elevated ambient temperatures (above the upper critical boundary of their thermoneutral zone), is to reduce the synthesis of T3 by switching the conversion of T4 to rT3 (reverse T3, an inactive form of T3) to reduce body heat production [57]. High environmental temperature also stimulates stress response, increasing heterophil/lymphocyte (H/L) ratio (a stress indicator), and suppresses immunity by reducing antibody production and altering the release of inflammatory factors such as cytokines [58,59]. Heat shock proteins (HSPs), such as HSP70, are important biomarkers in stress responses, including in HS and related inflammation—they control all aspects of cellular proteostasis, including protection of proteins from unfolding, aggregation, or denaturing to facilitate protein stabilization [60,61].

In our studies, H/L ratio, plasma levels of thyroid hormones (T4 and T3), cytokines (IL-6, IL-10, and interferon (IFN)- Y ), immunoglobulin (Ig) Y, and HSP70 were measured on the last day of the two chronic HS episodes (week 35 and 80, respectively) [43]. At week 35, the end of the first HS episode (weeks 21–35), CP hens had lower rectal temperatures (*p* < 0.05) than both AP and NP hens (Table 3). The CP hens also had lower HSP70 than NP hens (*p* < 0.05) but not AP hens (*p* > 0.05). At the end of the second HS episode (weeks 73–80), the CP hens had lower rectal temperatures (*p* < 0.05) and circulating H/L ratios (*p* < 0.01) than both AP and NP hens. The CP hens also had higher levels of T3 (*p* < 0.05) and T3/T4 ratios (*p* < 0.05) than NP hens but not AP hens, while CP hens had higher PCV (packed cell volume or hematocrit) levels than AP hens (*p* < 0.05) but not NP hens (*p* > 0.05). Cytokines and IgY levels were similar among treatments. These results indicate that the CP system has beneficial effects on physiological homeostasis and reduces the stress response of laying hens subjected to HS, particularly in aged hens (80-week-old). Of particular importance were the levels of thyroid hormones (T3 and T3/T4 ratio) in response to the second HS episode, which were in the order: CP > AP > NP. Generally, warm-blooded animals often reduce the synthesis of thyroid hormones to reduce body heat production and food intake in hot environments. These results indicate that CP hens were able to cope with HS better than NP and AP hens, with a lower HS-induced stress response and related cellular and tissue damage due to CP-transferred heat from hens’ feet to the chilled water.

### 3.5. Induced Molting under Hot Ambient Conditions

A commercial hen’s lifetime depends on the market egg demand, ranging from 75 to 110 weeks or longer with or without induced molting [13]. A hen experiences different stimuli (breeding program-, management practice-, and environmental-associated stressors) based on its life phases (duration as a chick, pullet, and layer with early, high, and low production stage), including HS, induced molting, and possible combinations during summer seasons, resulting in repeated and/or chronic stress responses. Induced molting brings the hens into a second laying cycle with improved egg and eggshell quality, reduced bird cost per dozen eggs, increased economic performance, and reduced number of male chicks to be euthanized [16]. Although induced molting may cause stress in hens [62], it remains a necessary management strategy to meet the egg demands since it is used as an effective intervention method to avoid interruptions in the egg market (egg supply shortages) whenever pullet shortages occur. Currently, only non-feed withdrawal molting processes—a diet offering low-energy ingredients such as wheat middlings—are allowed for those egg farms registered under the certified United Egg Producers husbandry welfare programs in the United States [13]. However, using low energy diets during molting may have negative effects on laying hens. For example, dietary manipulations may create an imbalance of nutrients [63,64]. Koch et al. [65] reported that non-feed withdrawal molted hens could still experience hunger, as indicated by increased feeding motivation. In general, the control of feeding is similar between chickens and mammals [18,66]; low energy diets may not lead to feelings of satiety even though the crop and proventriculus are distended following ingestion.

To meet uncontrollable market demands, molting may need to be induced during summer when high ambient temperatures are likely. The combined effects of heat and induced molting could be extra challenging for hens attempting to maintain or return to physiological homeostasis. In one of our studies, the goal was to examine the effects of the CP system on production performance and the health of caged laying hens subjected to induced molt under daily cyclic heat. Specifically, the objective of the following study was to investigate if providing the CP system to hens could alleviate the negative effects of daily cyclic heat and improve molting efficiency and post-molt performance.

In the study, the hens were subjected to a 28 d nonfasted molting regimen, starting at 85 weeks of age [67]. Cyclic heat of 32 °C (0600 to 1800 h) was applied daily during the molting period. After molt, hens were returned to a regular layer diet and housed under thermoneutral conditions until 110 weeks of age. Body weight (BW), rectal temperature, stress indicators (H/L ratio and corticosterone level), and thyroid hormones (T3 and T4) were measured during molting. Egg production and eggshell traits were measured during and after molting. Compared to NP and AP hens, CP hens had a higher feed usage and a greater BW loss, as well as lower H/L ratios (*p* < 0.05), with no difference in thyroid hormones and corticosterone at the end of molt (*p* > 0.05) (Table 4). Cooled perch hens also had higher egg production beginning at 98 weeks of age (*p*_*treatment* × *age*_ < 0.01) than NP hens and, occasionally, AP hens (Table 5). In addition, CP hens had higher rectal temperatures than NP hens (*p* < 0.01) but not AP hens (*p* > 0.05) at the end of molt (Table 5). These results indicate that CP hens were less stressed, evidenced by a lower H/L ratio with higher egg production, than NP and AP hens (Table 4). Likely, there are multiple reasons for the increased post-molt egg production of CP hens, especially that CP hens experienced faster and greater ovarian and oviduct regression during the molting process, as indicated by the greater BW loss, compared with NP hens (22.0% vs. 13.3%). Previous studies have evidenced that BW loss, approximately 25–30%, is a successful indicator of fasting molt [68], linked to catabolism of adipose, regression of the reproductive tract, and optimum post-molt egg production [69,70]. In addition, the greater feed usage suggests thermal and stress tolerance in CP hens, compared to NP and AP hens, during induced molting procedures combined with HS. These results indicate that the cooled perch can reduce the negative effects of combined induced molting and HS in laying hens, resulting in improved post-molt egg production.

## 4. Conclusions

Perches in laying hen facilities can be modified as alternative cooling devices. The cooled perch system facilitates hen thermoregulation under an elevated ambient temperature (35 °C) by transferring body heat from their feet to the cooled perches, ameliorating repeated or chronic HS with and without induced molt. Hens with access to the cooled perch system maintained thyroid hormonal homeostasis and related metabolism, immunity, and performance production under allostatic load. These results indicate that the thermal cooling system is a novel cooling method for improving the health, welfare, and production performance of laying hens during hot seasons, especially in tropical and subtropical regions.

## Figures and Tables

**Table 1:** The effects of cooled perches on hen mortality, production performance, and egg quality from 17 to 80 weeks of age.

Treatment ^1^	Cumulative Mortality (%)	Egg Production and Eggshell Quality ^2^					Feed Usage ^2^	
		Hen-Day Eggs (%)	Marketable Egg (%)	Egg Weight (g)	Breaking Force (N)	Eggshell Thickness (mm)	Feed Utilization (g Hen^−1^ d^−1^)	Feed Efficiency (kg Feed per Dozen Eggs)
NP	10.19 ^b^	72.6 ^c^	97.67	59.6 ^b^	35.0 ^b^	0.33	100.56 ^b^	1.84
AP	3.70 ^ab^	74.9 ^b^	97.80	60.0 ^b^	34.9 ^b^	0.33	98.28 ^b^	1.58
CP	2.78 ^a^	77.6 ^a^	98.26	61.1 ^a^	36.3 ^a^	0.34	103.02 ^a^	1.57
n ^3^	264	384	384	1320	1320	1320	264	264
SEM	1.93	0.5	0.23	0.1	0.3	0.002	0.82	0.12
*p* value								
*p_treatment_*	0.02	<0.0001	0.55	<0.0001	<0.0001	0.44	0.0002	0.23
*p_age_*	-	<0.0001	<0.0001	<0.0001	<0.0001	<0.0001	<0.0001	<0.0001
*p*_*treatment* × *age*_	-	<0.0001	0.17	<0.0001	0.01	0.33	0.04	0.19

^a–c^ Least square means within a column for the 3 treatments lacking a common superscript differ (*p* < 0.05). ^1^ AP, air perches; CP, cooled perches; and NP, control with no perch. ^2^ Values within a column represent the least square means of the samples from 17 to 80 weeks of age. ^3^ Average number of observations per least square means. (Modified from [43].)

**Table 2:** The effects of cooled perches on hen feather score and foot health at 80 weeks of age.

Treatment ^1^	Mean Feather Score ^1^	Foot Health ^2^		
		Hyperkeratosis Score ^2^	Mean Nail Length (cm)	Number of Broken Toenails
NP	1.95	3.83	1.8	1.13 ^ab^
AP	2.46	3.91	2.16	1.58 ^a^
CP	2.02	3.82	2.46	0.96 ^b^
n ^3^	24	24	24	24
SEM	0.27	0.24	0.23	0.18
*p* value	0.36	0.96	0.13	0.04

^a,b^ Least square means within a column for the 3 treatments lacking a common superscript differ (*p* < 0.05). ^1^ Scores for feather condition ranged from 1 to 4, with 4 representing no damage to the feathers and 1 representing severe damage. ^2^ Scores for hyperkeratosis ranged from 1 to 4, with 4 representing normal feet and 1 representing severe hyperkeratosis. ^3^ Average number of observations per least squares mean.

**Table 3:** The effects of cooled perches on plasma heterophil/lymphocyte ratio, immunological parameters, and thyroid hormones of caged laying hens.

Treatment ^1^	RT (°C)	PCV	H/L (ratio)	IL-6 (pg/mL)	IL-10 (pg/mL)	IFN-Υ (pg/mL)	IgY (ng/mL)	T3 (ng/mL)	T4 (μg/mL)	T3/T4 (ratio)	HSP70 (pg/mL)
First heating episode (35 weeks of age)											
NP	41.9 ^a^	27.1	0.81	34	62	72	340	184	6.44	0.029	294 ^b^
AP	41.9 ^a^	25.3	0.80	40	76	88	322	218	6.84	0.031	243 ^ab^
CP	41.7 ^b^	27.8	0.72	33	49	66	342	204	6.59	0.031	224 ^a^
n ^2^	24	24	24	24	24	24	24	24	24	24	24
SEM	0.10	1.2	0.08	4	7	12	28	9	0.12	0.003	20
*p*-value	0.02	0.34	0.48	0.54	0.08	0.40	0.86	0.11	0.07	0.13	0.04
Second heating episode (80 weeks of age)											
NP	41.4 ^a^	30.3 ^ab^	1.24 ^a^	25	45	24	214	245 ^b^	7.74	0.032 ^b^	234
AP	41.4 ^a^	29.4 ^b^	1.20 ^a^	25	43	48	168	269 ^ab^	7.88	0.034 ^ab^	215
CP	41.1 ^b^	31.3 ^a^	0.83 ^b^	29	39	48	157	288 ^a^	7.69	0.038 ^a^	166
n ^2^	24	24	24	24	24	24	24	24	24	24	24
SEM	0.15	0.4	0.08	4	7.5	7	37	12	0.17	0.001	23
*p*-value	0.02	0.02	0.01	0.78	0.78	0.43	0.51	0.002	0.48	0.0006	0.10

^a,b^ Least square means within a column for the 3 treatments lacking a common superscript differ (*p* < 0.05). ^1^ AP, air perches; CP, cooled perches; and NP, control with no perch. ^2^ Average number of observations per least square means. (Modified from [44].) H/L, heterophil/lymphocyte ratio; HSP 70, heat shock protein 70; IFN-Υ, interferon-Υ; IgY, immunoglubin Y; IL, interleukin; PVC, packed cell volume; RT, rectal temperature; T3, triiodothyronine; and T4, thyroxin.

**Table 4:** The effects of cooled perches on rectal temperature (RT), body weight, feed intake, stress indicators (H/L, CORT), and thyroid hormones in caged laying hens during induced molt combined with daily cyclic heat or immediately post-induced molt.

Treatment ^1^	RT ^2^ (°C)	Body Weight			Feed Intake (g Hen^−1^ d^−1^)	H/L (Ratio)	CORT (ng mL^−1^)	T3 (ng mL^−1^)	T4 (μg mL^−1^)	T3/T4 (Ratio)
		Initial (kg)	Post-Molting (kg)	Loss (%) ^3^						
NP	41.9 ^a^	1.57	1.35	13.3 ^b^	53.7 ^b^	1.38 ^a^	6.47	230	4.70	0.051
AP	41.8 ^ab^	1.60	1.27	19.8 ^ab^	52.6 ^b^	1.62 ^a^	6.27	213	4.84	0.047
CP	41.6 ^b^	1.58	1.25	22.0 ^a^	58.6 ^a^	0.91 ^b^	4.71	223	4.81	0.048
SEM	0.12	0.05	0.04	2.2	1.5	0.08	0.86	10	0.17	0.003
n ^4^	24	24	24	24	24	24	24	24	24	24
*p*-value	0.01	0.94	0.11	0.02	0.02	0.01	0.38	0.53	0.82	0.71

^a,b^ Least square means within a column for the 3 treatments lacking a common superscript differ (*p* < 0.05). ^1^ AP, air perches; CP, cooled perches; and NP, control with no perch. ^2^ RT (rectal temperature) was taken from 2 birds per cage at 28 days immediately post-molt when hens were still exposed to heat. ^3^ Percentage of body weight loss: (initial body weight—28-day body weight)/initial body weight × 100. ^4^ Average number of observations per least square means. (Modified from [67].)

**Table 5:** The effects of cooled perches on egg production and eggshell traits of caged laying hens post-molt from 89–110 weeks of age.

Treatment ^1^	50% Egg Production (d)	Hen-Day Egg Production ^1^ (%)	Egg Weight ^2^ (g)	Breaking Force ^2^ (N)	Proportion of Eggshell ^2^ (%)	Eggshell Thickness ^2^ (mm)
NP	47.6	51.4	69.3	35.4	8.67 ^a^	0.350
AP	48.2	54.4	69.7	33.2	8.32 ^b^	0.341
CP	48.8	59.3	69.9	34.3	8.62 ^a^	0.347
SEM	0.7	2.8	0.5	1.2	0.09	0.001
n ^3^	12	264	240	240	240	240
*p*-value						
p_treatment_	0.49	0.29	0.66	0.45	0.03	0.09
p_age_	-	<0.0001	<0.0001	<0.0001	0.04	0.04
p_treatment × age_	-	<0.0001	0.01	0.50	0.61	0.60

^a,b^ Least square means within a column for the 3 treatments lacking a common superscript differ (*p* < 0.05). ^1^ AP, air perches; CP, cooled perches; and NP, control with no perch. ^2^ The least square means averaged over 22 weeks of post-molt egg production and eggshell traits (89–110 weeks of age). ^3^ Average number of observations per least square means: 12 cages, and accumulated 264 hens and 240 eggs per treatment, respectively. (Modified from [67].)

## Data Availability

Data are contained within the article.
